# Epidemiological characteristics of adenovirus in children in Yancheng, China, 2023-2024

**DOI:** 10.3389/fcimb.2025.1587257

**Published:** 2025-05-29

**Authors:** Guoqing Chen, Jin Cao, Yingli Qu, Wenyan Tian, Peng Shen, Xuan Liu, Qin Luo, Qinqin Song, Qi Wen, Guangyu Xue, Yuting Hu, Qiangqiang Shi, Lifeng Zhang, Rundong Zhu, Juan Song, Jun Han

**Affiliations:** ^1^ Key Laboratory of Molecular Biology in Public Health, Yancheng Center for Disease Prevention and Control, Yancheng, China; ^2^ National Key Laboratory of Intelligent Tracking and Forecasting for Infectious Diseases, National Institute for Viral Disease Control and Prevention, Chinese Center for Disease Control and Prevention, Beijing, China; ^3^ School of Medicine, Anhui University of Science and Technology, Huainan, China

**Keywords:** respiratory infections, adenovirus, epidemiological characteristics, genetic features, phylogenetic trees

## Abstract

**Objective:**

To understand the epidemiology and evolutionary characteristics of human adenovirus (HAdV) infecting children in Yancheng, China, during the winter of 2023-2024.

**Methods:**

HAdV-positive throat swab samples were collected from pediatric patients in Yancheng. The Hexon, Penton base, and Fiber genes were amplified and sequenced for typing and phylogenetic analysis of HAdV.

**Results:**

170 HAdV-positive samples were collected and identified from children with acute respiratory infection (ARI) in Yancheng. A total of 133 samples were successfully amplified and sequenced for Hexon, Penton base, and Fiber genes. Phylogenetic analysis showed that HAdV-B3 was the dominant circulating strain, and two strains of B21, one strain of C1 and one strain of C5 were also identified.

**Conclusion:**

During the winter of 2023-2024, HAdV infecting children in Yancheng was mainly the B3 type.

## 1 Introduction

Acute respiratory infection (ARI) is a clinical acute disease caused by various pathogens with acute respiratory symptoms (usually lasting no more than 21 days) (Chretien et al., 1984). The main symptoms include cough, sputum production, shortness of breath, sore throat, and runny nose. ARI primarily includes acute upper respiratory tract infections, acute bronchitis, and community-acquired pneumonia (CAP), with common pathogens being viruses and bacteria (including atypical pathogens). Upper respiratory tract infections can occur year-round, with a higher incidence during winter and spring. Most cases are self-limiting, but the elderly, children, those with underlying diseases, and immunocompromised individuals are at risk of developing bronchitis, pneumonia, severe pneumonia, and even life-threatening conditions. Approximately 4 million people reportedly die from respiratory viral infections each year, particularly the elderly, children, and immunocompromised populations (Avendaño Carvajal and Perret Pérez, 2020). In ARI, upper respiratory tract infections account for 70–90% of cases, with 70–80% caused by viruses. Common viruses include influenza virus, rhinovirus, coronavirus, parainfluenza virus, respiratory syncytial virus, adenovirus, human metapneumovirus, etc (Weber et al., 1998; Chang et al., 2002; Huijskens et al., 2012). HAdV is one of the common pathogens that cause ARI (Wang et al., 2024). The main manifestation is acute upper respiratory tract infection (2% to 4% of acute respiratory tract infections are caused by adenovirus), followed by eye and gastrointestinal infections. HAdV can infect people of all ages. ARI caused by HAdV accounts for 1-10% in adults and 5-10% in children (Kenmoe et al., 2018). The clinical manifestations of primary HAdV infection are mostly subtle (Yu et al., 2021), and the virus can be latent in mucosal lymphocytes, such as tonsils and adenoids after infection (Garnett et al., 2009). However, reactivation of latent HAdV in neonates, the elderly, or immunocompromised individuals can lead to more severe respiratory disease (Lynch et al., 2011).

The HAdV genome is 34 to 36 kb in length (Ismail et al., 2018) and is an unenveloped, double-stranded DNA virus with a diameter of 70 to 100 nm (Shieh, 2022). It has a spherical particle structure with icosahedral symmetry, consisting of 240 hexon proteins and 12 penton proteins attached to trimeric fibers of various sizes at the 12 vertices (Lion, 2014). The hexon protein is the main antigenic protein of HAdV, and the presence of a hypervariable structural loop in this region contains specific antigen-determining sites, allowing HAdV to be preliminarily typed by analyzing the hexon protein (Madisch et al., 2005). Specific antigen-determining sites are also present on the fiber protein, which, together with the hexon protein determines the genotyping of HAdV (Zou et al., 2021; Pan et al., 2023). Although the specificity of the penton base protein is weak (Lynch et al., 2011), the current typing of HAdV is based on the combination of hexon, penton base, and fiber due to the recombination of HAdV (Wu et al., 2022).

According to their biological and immunological characteristics, HAdV is divided into seven groups (A-G) and more than 100 genotypes (Rowe Wp et al., 1953; Walsh et al., 2009). The tissue and organ tropism of different HAdV groups varies (SSan Martín, 2012), with respiratory infections primarily caused by groups B (HAdV-3, 7, 11, 14, 16, 21, 50, 55), C (HAdV-1, 2, 5, 6), and E (HAdV-4) (Dou et al., 2018; Yao et al., 2019). Adenovirus pneumonia accounts for approximately 4% -10% of community-acquired pneumonia, and severe pneumonia is more common in HAdV-3 and HAdV-7 (Xie et al., 2018). HAdV-55, a recombination of adenovirus types 11 and 14, causes multiple outbreaks (Lu et al., 2014). HAdV-21 is associated with severe disease, rapidly progressing to multi-organ failure and death (Pfortmueller et al., 2019). The prevalence of HAdV exhibits distinct characteristics influenced by climate, environment, and region (Brini et al., 2020). Outbreaks and epidemics of HAdV frequently occur in highly confined and crowded environments, such as military barracks, hospitals, and schools (Tamura et al., 2007). Since its discovery in 1953, HAdV outbreaks have been reported globally, including in China, indicating that adenovirus outbreaks remain relatively common. Recent reports highlight adenovirus variants with increased pathogenicity, as well as cases of zoonotic transmission and interspecies recombination (Lange et al., 2019). Adenoviruses still pose a threat to public health.

In recent years, ARI caused by newly emerging HAdV strains has been frequently reported worldwide. At present, there is no effective vaccine or antiviral drug for HAdV infection, therefore, monitoring the variation of HAdV strains and the emergence of new viruses is very important. This study aims to provide valuable genomic data and elucidate the molecular evolutionary characteristics of HAdV. In this study, Hexon, Penton base, and Fiber genes were successfully amplified and sequenced from 133 HAdV-positive samples. Phylogenetic analysis showed that 129 strains belonged to genotype B3, two strains to B21, and one strain each to C1 and C5, indicating that B3 was the predominant circulating strain in Yancheng during the winter of 2023-2024. In addition, molecular evolutionary features of HAdV were depicted by analyzing nucleotide identity and amino acid variation sites.

## 2 Materials and methods

### 2.1 Sample

The samples were collected from October 2023 to March 2024 at national influenza sentinel surveillance hospitals (Yancheng First People’s Hospital and Yancheng Third People’s Hospital) from specimens diagnosed as influenza-like illness (ILI) cases but tested negative for influenza virus. A total of 827 cases were randomly selected, of which 170 cases from pediatric patients were positive for HAdV, yielding a positivity rate of 20.4%. These samples were transported by dry ice throughout the cold chain to National Institute for Viral Control and Prevention, Chinese Center for Disease Control and Prevention for further identification.

### 2.2 Sample processing and nucleic acid extraction

The throat swab samples were thawed at room temperature, divided into sterile cryopreservation tubes, and 200μL of each sample was used for nucleic acid extraction. Nucleic acids extraction was performed using the automated nucleic acid extractor (GeneRotex96, Tianlong, China) and the corresponding nucleic acid extraction kit (ZTLJB-Y64T), following the manufacturer’s instructions. The extracted nucleic acids were stored at 4 °C for subsequent experiments.

### 2.3 Detection of real-time qPCR

The primers and probe used for HAdV detection were as follows: forward primer 5’-GCCACGGTGGGGTTTCTAAACTT-3’, reverse primer 5’-GCCCCAGTGGTCTTACATGCACATC-3’, and probe 5’-TGCACCAGACCCGGGCTCAGGTACTCCGA-3’. They were designed and validated in-house, and synthesized by Shanghai Bioengineering Co., Ltd. 2μl nucleic acid template was added to the reaction system of real-time PCR according to the kit’s instructions (the GoldStar Probe Mixture Real-Time PCR Kit, China) and amplified for 35 cycles using the ABI QuantStudio 5.

### 2.4 Amplification of Hexon, Penton base, and Fiber gene

The primers for amplifying the Hexon, Penton base, and Fiber genes were designed according to a previous report (**Table 1**) (Wu et al., 2022). PCR conditions were determined, and reactions were conducted in a total volume of 50 μl comprising the Vazyme 2× Phanta Max Master Mix (25 μl), primer F (10 μmol/L, 2 μl), primer R (10 μmol/L, 2 μl), DNA template (3 μl), and water (18 μl). PCR reaction procedure: Initial denaturation for 3 minutes at 95 °C, followed by 15 seconds at 95 °C, 15 seconds at 56 °C, and 60 seconds at 72 °C for a total of 35 cycles, and finally extended for 5 minutes at 72 °C. The PCR products were subjected to Sanger sequencing (Qingke Biosciences Co., Ltd. China) for subsequent analysis. The sequences generated in this study were submitted to GenBank with accession numbers from PV468604 to PV468693.

**Table 1:** Universal primers for the typing and sequencing of HAdV.

Gene	Position	Primer	Primer sequence	Position	Length(bp)
Hexon	13,904–15,538	HVR-F	5'-CAGGATGCTTCGGAGTACCTGAG-3'	14,152–14,175	1,253bp
		HVR-R	5'-TTTCTGAAGTTCCACTCGTAGGTGTA-3'	15,384–15,404	
Penton base	18,422–21,256	Penton-F	5'-CTATCAGAACGACCACAGCAACTT-3'	18,473–18,495	1,685bp
		Penton-R	5'-TCCCGTGATCTGTGAGAGCRG-3'	20,132–20,157	
Fiber	31,301–32,260	Fiber-F	5'-CCCTCTTCCCAACTCTGGTA-3'	31,180–31,199	1,153bp(B)/1,519bp(E)
		Fiber-R	5'-GGGGAGGCAAAATAACTACTCG-3'	32,311–32,332	
		Fiber-CR	5'-GAGGTGGCAGGTTGAATACTAG-3'	32,311–32,332	2,027bp(C)

### 2.5 Sequence analysis

The sequenced fragments were assembled using SeqMan 7.1.0 (DNASTAR), and the assembled sequences were subjected to BLAST (Basic Local Alignment Search Tool) on NCBI (National Center for Biotechnology Information) to verify successful amplification. Prototype strain sequences and reference sequences from various regions and genotypes were downloaded from the NCBI GenBank database. These sequences were aligned using Clustal W within MEGA 7.0. Phylogenetic analyses were conducted using the aligned sequences with the Maximum Likelihood method in MEGA 7.0, employing the best-fit substitution model for each alignment. A bootstrapping analysis of 1,000 replicates was performed, and bootstrap values exceeding 60% were indicated on the trees. The phylogenetic trees were further beautified online using ITOL (https://itol.embl.de). MegAlign software was used to analyze the identity of nucleotides and amino acids of the sequences. The amino acid variant sites were analyzed using MEGA 7.0 software.

### 2.6 Recombination analysis

Potential recombination events were screened using RDP5 software (Martin et al., 2015). The putative recombinant sequences identified by RDP5 were subsequently validated with Simplot 3.5.1.4. A sliding window size of 200 bp with a step size of 20 bp was employed (Lei et al., 2023).

### 2.7 Statistical analysis

Statistical analysis was performed using SPSS 27 software, and the differences in each clinical symptom between HAdV-positive and HAdV-negative patients were compared using the chi-square test. Differences were considered significant when the *p* -value was below 0.05.

## 3 Results

### 3.1 Demographic and clinical information

In this study, we collected a total of 170 HAdV-positive samples from 827 influenza-negative ILI cases. Of these HAdV-positive cases, 108 (63.5%) were male and 62 (36.5%) were female, yielding a male-to-female ratio of 1.74:1. The patients’ ages ranged from 2 to 14 years, with a median age of 6.5 years. The highest detection rate of HAdV was in January 2024 (49%, 83/170), followed by December 2023 (24%, 41/170).

The main clinical symptoms of the HAdV-positive patients were fever (100%, 170/170), cough (54%, 92/170), sore throat (28%, 47/170), and body aches (6%, 11/170). Mixed clinical presentations were observed, including concurrent cough and sore throat (22%, 38/170), cough with body aches (4%, 7/170), as well as sore throat with body aches (2%, 4/170). All cases had a fever, with a temperature range of 37.4-41.5 °C. Compared to 657 non-HAdV cases, those with similar main clinical symptoms: fever (99%, 652/657), cough (36%, 235/657), sore throat (13%, 88/657), and body aches (4%, 30/657). Each clinical symptom between HAdV-positive patients and HAdV-negative patients was analyzed using the chi-squared test (**Supplementary Table S1**). The results showed that the incidence of both cough and sore throat in HAdV-positive patients is significantly higher than that in HAdV-negative patients (p <0.05).

### 3.2 Grouping of HAdV

**Figure 1:**
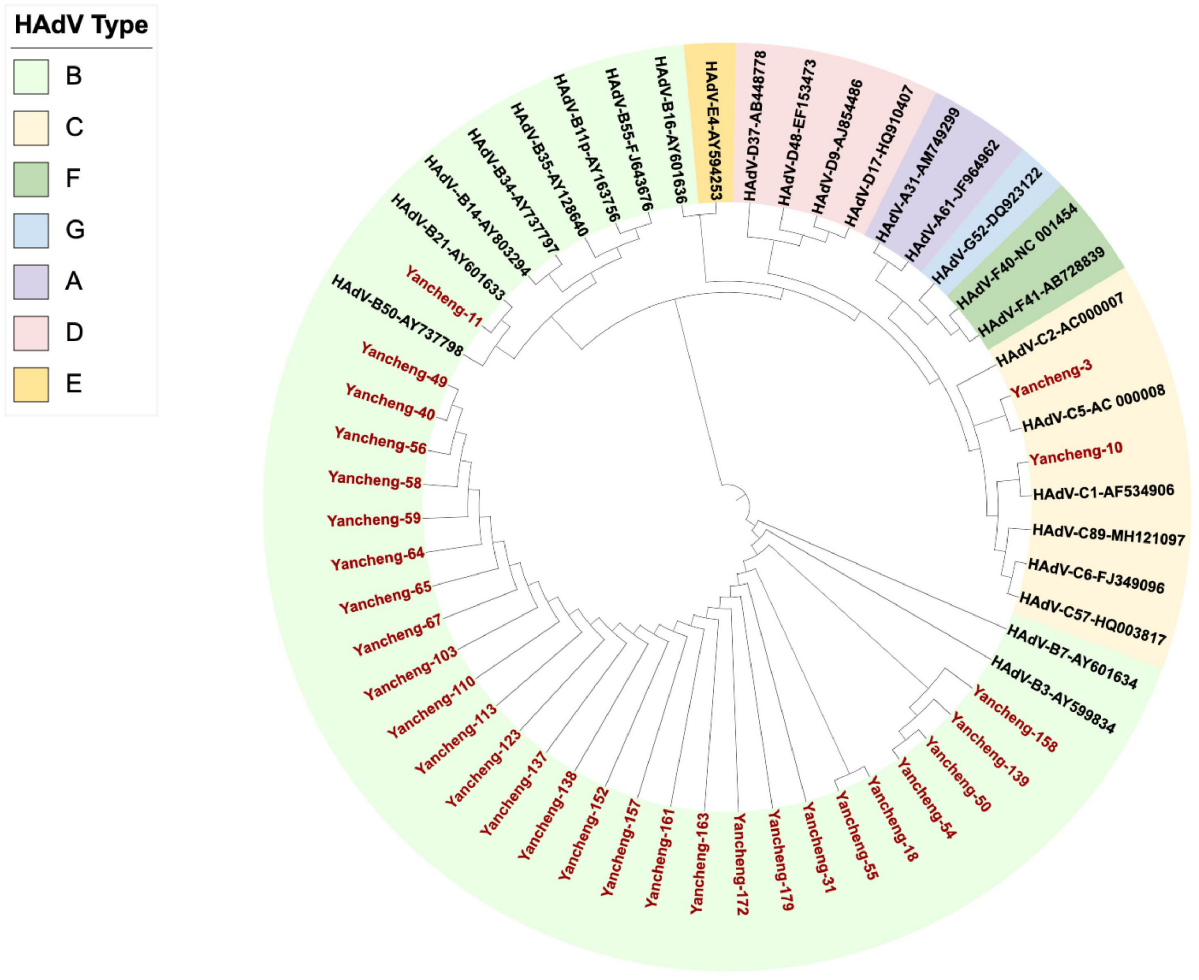
Phylogenetic analysis of Hexon gene in Yancheng from 2023 to 2024. (The red font indicates the sample of this study, and the others are the prototype strains of each type.).

133 of Hexon, Penton base and Fiber genes were successfully amplified and sequenced from 170 positive samples. Due to the large sample size, 103 specimens with completely identical sequences of all three target genes (Hexon, Penton base, and Fiber) were considered as one sample, and finally 30 unique samples were screened for analysis. The phylogenetic tree was constructed with Hexon gene sequences of this study combined with the prototype strains from each HAdV group (**Figure 1**). The results showed that 27 strains of B3, one strain of B21, one strain of C5, and one strain of C1 were identified on the phylogenetic tree of the Hexon gene. The nucleotide identity of the Hexon gene among the 27 B3 strains ranged from 99.4% to 100%. For HAdV-C5 (Yancheng-3) and HAdV-C1 (Yancheng-10), the nucleotide identity of the Hexon gene was 77.6%.

### 3.3 Evolutionary analysis of HAdV B isolates

#### 3.3.1 Phylogenetic analysis

**Figure 2:**
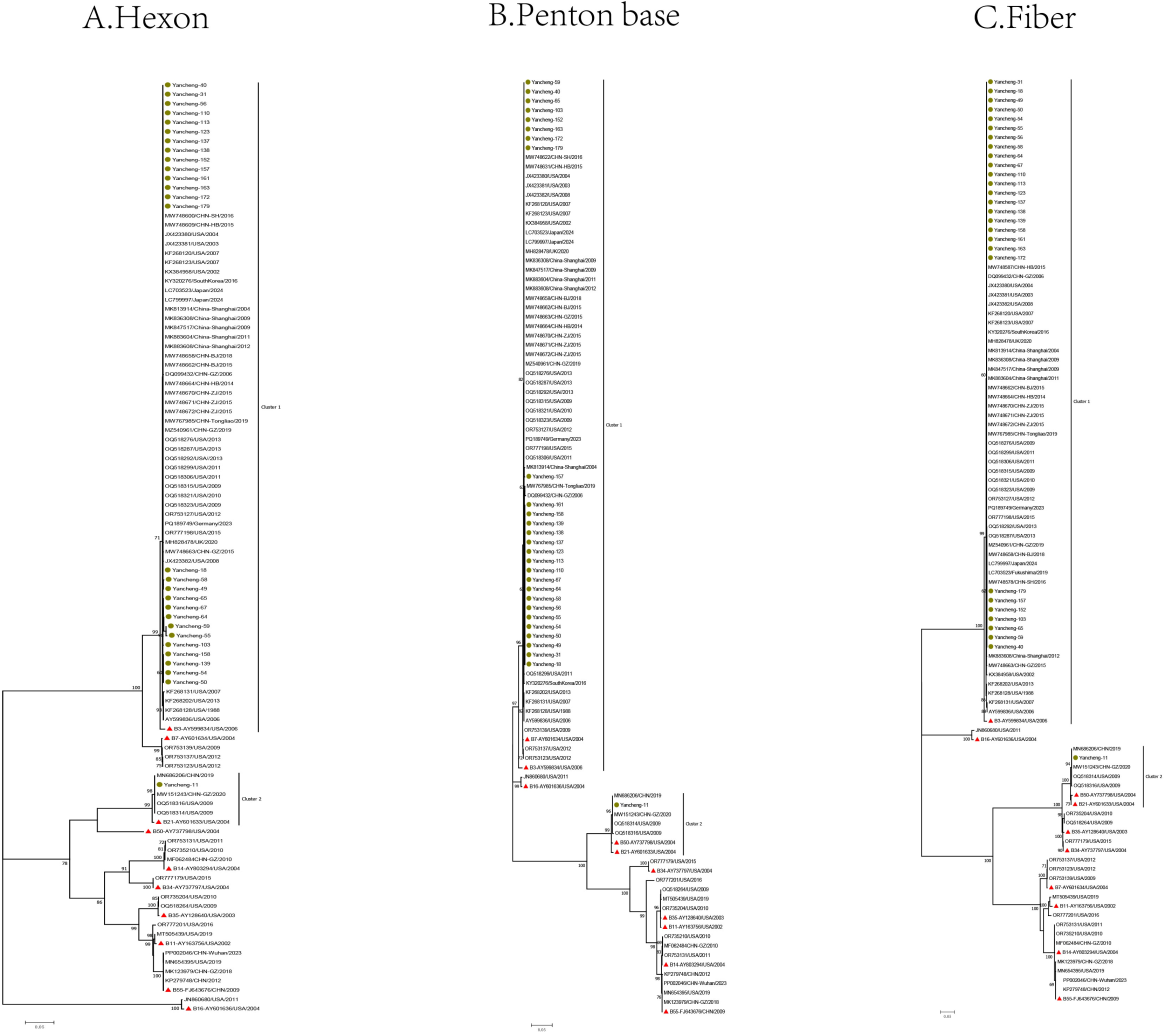
Phylogenetic analysis of Hexon **(A)**, Penton base **(B)**, and Fiber **(C)** genes of HAdV-B samples in Yancheng from 2023 to 2024 (The green circle represents the sequence of this study, the red triangle represents the prototype strain of each type, and the rest are reference sequences for each type).

In this study, one of the identical sequences was selected for subsequent analysis. The Hexon, Penton base, and Fiber gene sequences of the 28 type B HAdV strains in this study, along with the reference sequences of species B, were used to construct phylogenetic trees (**Figure 2**).

In the phylogenetic tree of the Hexon gene, the B21 strain (Yancheng-11) formed a cluster with four reference sequences from the United States, Guangzhou and Nanjing (China), and the prototype B21 strain AV-1645 (AY601633) from the United States. Type B3 strains (Yancheng-18, 31, 40, 49, 50, 54, 55, 56, 58, 59, 64, 65, 67, 103, 110, 113, 123, 137, 138, 139, 152, 157, 158, 161, 163, 172, and 179) clustered with reference sequences from the United States, Germany, Japan, South Korea, the UK, and China (Beijing, Hebei, Guangzhou, Shanghai, Zhejiang, and Tongliao), along with the prototype B3 strain GB (AY599834). The remaining reference sequences formed other branches.

In the Penton base gene, Yancheng-11 still formed a cluster with above four reference sequences from the United States, China (Guangzhou and Nanjing), the prototype B21 strain AV-1645 (AY601633), additionally, the prototype B50 strain Wan (AY737798) was grouped within the B21 cluster. Yancheng-18, 31, 40, 49, 50, 54, 55, 56, 58, 59, 64, 65, 67, 103, 110, 113, 123, 137, 138, 139, 152, 157, 158, 161, 163, 172, and 179 were still consistent with reference sequences from the United States, Germany, Japan, Korea, UK, and China (Beijing, Hebei, Guangzhou, Shanghai, Zhejiang, and Tongliao), as well as the prototype B3 strain GB (AY599834) from the United States. Additionally, the prototype American B7 strain NHRC-1315 (AY601634) and three U.S.-derived reference sequences were grouped within the B3 cluster.

In the Fiber gene, the branches formed by Yancheng-11 were consistent with those of the Penton base gene in the phylogenetic tree. Similarly, the branches of Yancheng-18, 31, 40, 49, 50, 54, 55, 56, 58, 59, 64, 65, 67, 103, 110, 113, 123, 137, 138, 139, 152, 157, 158, 161, 163, 172, and 179 strains were consistent with those of the Hexon gene in the tree.

The results of phylogenetic analysis suggested that there was no recombination event of the HAdV-B strain prevalent in Yancheng in this study. Most of the HAdV-B3 strains in this study clustered on the same branch as the prototype strain of B3 in the Hexon, Penton, and Fiber evolutionary trees (**Figure 2**), indicating that they may come from a common ancestor. Similarly, the B21 strain (Yancheng-11) formed monophyletic branches with the prototype strain B21 in the Hexon, Penton and Fiber gene trees, indicating that they may originated from a common ancestor. The HAdV-B3 exhibits a global distribution pattern, forming a well-supported cluster with reference strains from the United States, Germany, Japan, South Korea, the UK, and multiple provinces in China (Beijing, Hebei, Guangzhou, Shanghai, Zhejiang, and Tongliao). This indicates that the B3 strain may have strong adaptability to transmission.

#### 3.3.2 Nucleotide and amino acid identities

The 28 HAdV-B sequences were analyzed for identity using MegAlign (**Supplementary Figure S2**). For the 28 HAdV-B strains, the identity of nucleotides and amino acids of the Hexon gene ranged from 72.2% to 100% and 62.9% to 100%, respectively. For the Penton base gene, the identity of nucleotides and amino acids ranged from 81.3% to 100% and 83.7% to 100%, respectively. For the Fiber gene, the identity of nucleotides and amino acids ranged from 61.5% to 100% and 55.7% to 100%, respectively (**Supplementary Table S3**).

For the Hexon gene, the identity of nucleotides and amino acids between the B21 strain from Yancheng-11 and the prototype strain AV-1645 were 99.1% and 98.1%. The identity of nucleotides and amino acids among the 27 B3 strains in this study ranged from 99.4% to 100% and 99.0% to 100%; the identity of nucleotides and amino acids between the 27 B3 strains and the prototype strain GB ranged from 98.3% to 100% and 96.9% to 100%.

For the Penton base gene, the identity of nucleotides and amino acids between the B21 strain from Yancheng-11 and the prototype strain AV-1645 were 94.5% and 93.7%. The identity of nucleotides and amino acids among the 27 B3 strains in this study ranged from 99.8% to 100% and 99.0% to 100%; the identity of nucleotides and amino acids between the 27 B3 strains of this study and the prototype strain GB ranged from 98.0% to 100% and 98.9% to 100%.

For the Fiber gene, the identity of nucleotides and amino acids between the B21 strain from Yancheng-11 and the prototype strain AV-1645 were 99.4% and 99.1%. The identity of nucleotides and amino acids among the 27 B3 strains of this study ranged from 94.0% to 100% and 93.8% to 100%; the identity of nucleotides and amino acids between the 27 B3 strains of this study and the prototype strain GB ranged from 92.6% to 100% and 93.1% to 100%.

The analysis of both nucleotide and amino acid identities revealed distinct evolutionary patterns among the HAdV-B strains. The B3 strains exhibited exceptionally high sequence conservation (Hexon gene amino acid identity: 99.0-100%), suggesting potential clonal propagation within the local population. In contrast, the B21 strain showed relatively lower identity with prototype strain AV-1645 in the Penton base gene (93.7% amino acid identity), indicating this region may be under strong selective pressure. Notably, the Fiber gene demonstrated the highest variability (amino acid identity range: 55.7-100%), consistent with its functional role in receptor binding and antigenic diversification (Liu et al., 2023).

#### 3.3.3 Analysis of amino acids variant sites

Subsequently, amino acid variation sites of three target genes were analyzed between the B21 HAdV strain in this study and the prototype strain AV-1645. The results showed that 9, 4, and 3 amino acid sites were mutated in the Hexon, Penton base, and Fiber genes, respectively. These mutations included D144N, G146E, T216A, P256L, E272Q, I423T, S424T, Q434E, and F455S in the Hexon gene; A335G, D353E, K378R, and S379G in the Penton base gene; and N49K, I70T, and R279H in the Fiber gene. The differences in the three target genes were compared among 27 HAdV-B3 strains in this study and the prototype GB strain. The results showed that 15, 4, and 10 amino acid sites were mutated in the three target genes, respectively. These mutations included G141R/Q, E262D, D265E/K, G266E, R267K, D268G/N/E, A269F, E299G, N302D, N411D, T418R, T429A, A439D, P440T, and T445A in the Hexon gene; T158I, K171T, I178T, and D326N in the Penton base gene; S22N, S23L, D72N, Q150E, S207L, E215Q, A222T, D223H, H246D, and M272T in the Fiber gene.

Whether HAdV-B3 strains or the HAdV-B21 strain, some mutations were located in the hypervariable regions (HVRs) of the Hexon protein, which are critical areas for the interaction between the virus and the host immune system. Mutations such as D144N and G146E, D265E/K and G266E may alter the charge distribution of HVRs, affecting the virus’s ability to bind to antibodies. G141R/Q and R267K may affect the structural stability of Hexon protein, indirectly affecting its binding ability to receptors. Mutations such as A269F and T445A may increase the persistence of the virus in the host’s body, thereby improving its transmission efficiency (Crawford-Miksza and Schnurr, 1996; Arnberg et al., 2000).

### 3.4 Phylogenetic analysis of HAdV C isolates

#### 3.4.1 Phylogenetic analysis

**Figure 3:**
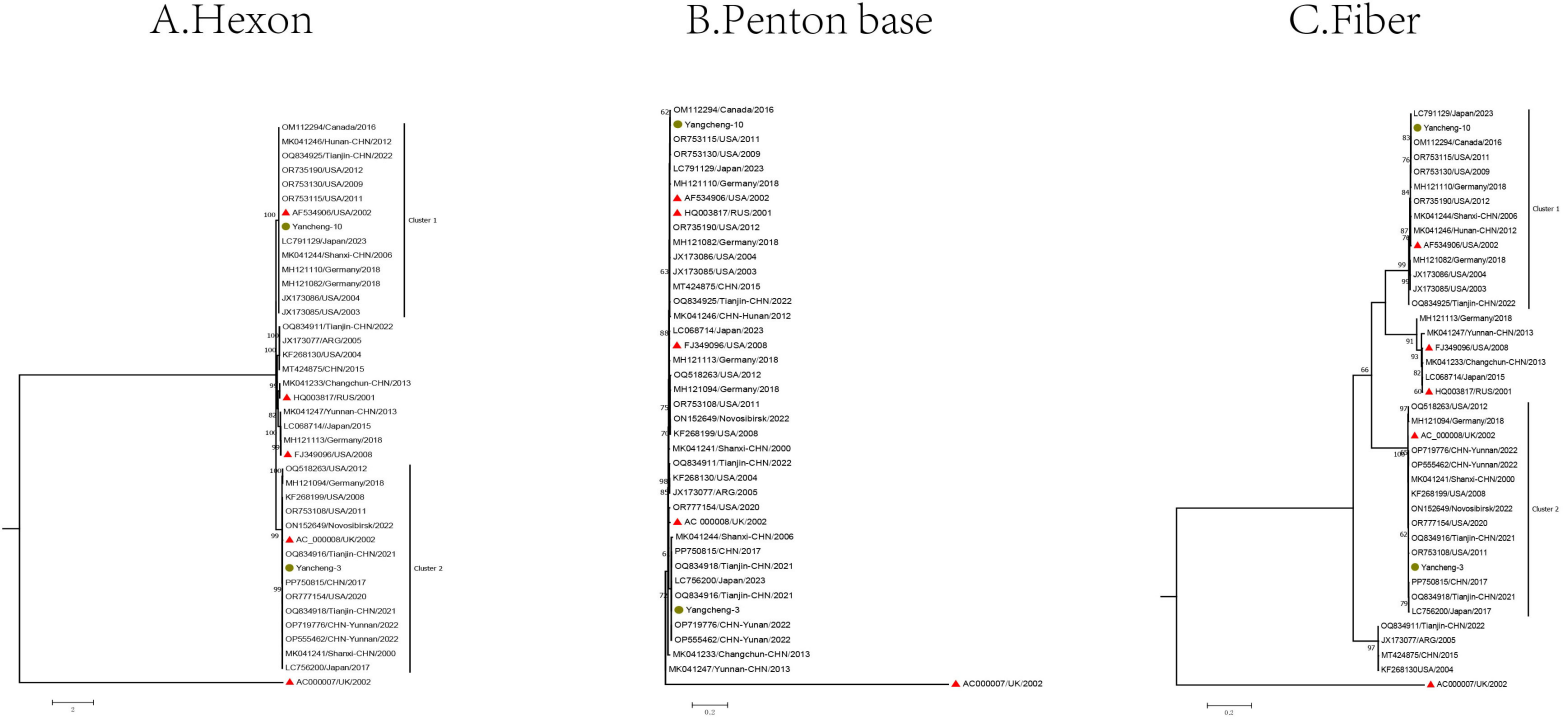
Phylogenetic analysis of two HAdV-C samples in Yancheng from 2023 to 2024 based on Hexon **(A)**, Penton base **(B)**, and Fiber **(C)** genes (The yellow circle is the study sequence, the red triangle is the prototype strain of each type, and the rest is the reference sequence of each type).

The Hexon, Penton base, and Fiber gene sequences of the two type C HAdV strains, along with the reference sequences of species C, were used to construct phylogenetic trees (**Figure 3**).

In the Hexon gene phylogenetic analysis, two species C strains (Yancheng-3 and Yancheng-10) of this study exhibited distinct clustering patterns. Yancheng-3 clustered with reference sequences from the United States, Germany, the UK, Japan, and China (Tianjin, Yunnan, and Shanxi), along with the US prototype strain C5 (AC_000008). In contrast, Yancheng-10 formed a separate cluster comprising reference sequences from Germany, Japan, Canada, and China (Tianjin, Hunan, Shanxi), grouping with prototype strain C1 (AF534906).

In the Fiber gene, the branches formed by Yancheng-3 and Yancheng-10 were similar to those of the Hexon gene. In the Penton base gene, the branches diverged from those of the Hexon and Fiber genes due to smaller genetic distances between types.

Phylogenetic analysis revealed no evidence of major recombination events among the HAdV-C strains circulating in Yancheng. Two HAdV-C strains clustered with reference sequences from multiple Chinese provinces (Tianjin, Yunnan, and Shanxi).

#### 3.4.2 Nucleotide and amino acid identities

In this study, two HAdV-C sequences (Yancheng-3 and Yancheng-10) were analyzed for identity using MegAlign. For the two C strains, nucleotide and amino acid identities of the Hexon gene were 77.6% and 76.2%, respectively. For the Fiber gene, nucleotide and amino acid identities were 75.6% and 50.4%, respectively. For the Penton base gene, nucleotide and amino acid identities were 96.1% and 96.8%, respectively, and the identity of nucleotides and amino acids among all species C ranged from 96.1% to 100% and 96.2% to 100%.

For the Hexon gene, the identity of nucleotides and amino acids between Yancheng-3 and the prototype C5 strain (AC_000008) were 99.2% and 99.6%, respectively. The identity of nucleotides and amino acids between Yancheng-10 and the prototype C1 strain (AF534906) were 99.9% and 99.9%, respectively.

For the Fiber gene, the identity of nucleotides and amino acids between Yancheng-3 and the prototype C5 strain (AC_000008) were 99.9% and 99.8%. The identity of nucleotides and amino acids between Yancheng-10 and the prototype C1 strain (AF534906) were 99.5% and 99.0%.

For the Penton base gene, the identity of nucleotides and amino acids between Yancheng-3 and the prototype C5 strain (AC_000008) were 97.6% and 98.4%. The identity of nucleotides and amino acids between Yancheng-10 and the prototype C1 strain (AF534906) were 99.4% and 99.2%.

The HAdV-C1 and HAdV-C5 strains from Yancheng exhibited high amino acid identity (98.4-99.9%) compared to their respective prototype strains, indicating limited genetic divergence during local evolution. This conservation pattern suggests strong purifying selection acting on these HAdV-C strains in Yancheng, with only minor region-specific variations observed.

#### 3.4.3 Analysis of amino acids variant sites

The differences were compared among the Hexon, Penton base, and Fiber genes of C5 strain from Yancheng-3 and the prototype strain AC_000008. Two amino acid variation sites were found on Hexon gene including I144M and T273A; Four amino acid variation sites were found on Penton base gene including L152S, P153H, S310G, and A363D. However, nucleotide differences in the Fiber gene did not lead to amino acid mutations (nonsense mutations). Compared with C1 prototype strain AF534906, there were also nonsense mutations on the Hexon of C1 strain of Yancheng-10; D277N, A327P, and P364L mutations on Penton base; and K74E, N199S, R442K, and E527D mutations on Fiber.

### 3.5 Recombination analysis

Potential recombination events in the Fiber gene were respectively identified in strains Yancheng-10 and Yancheng-11 using RDP5 software. The Fiber gene of C1 strain Yancheng-10 detected recombination sources including the major parent strain C5-MH121094 and the minor parent strain C6-MH121113. The Fiber gene of B21 strain Yancheng-11 detected recombination sources including the major parent strain B3-AY599836 and the minor parent strain B11-AY163756.

**Figure 4:**
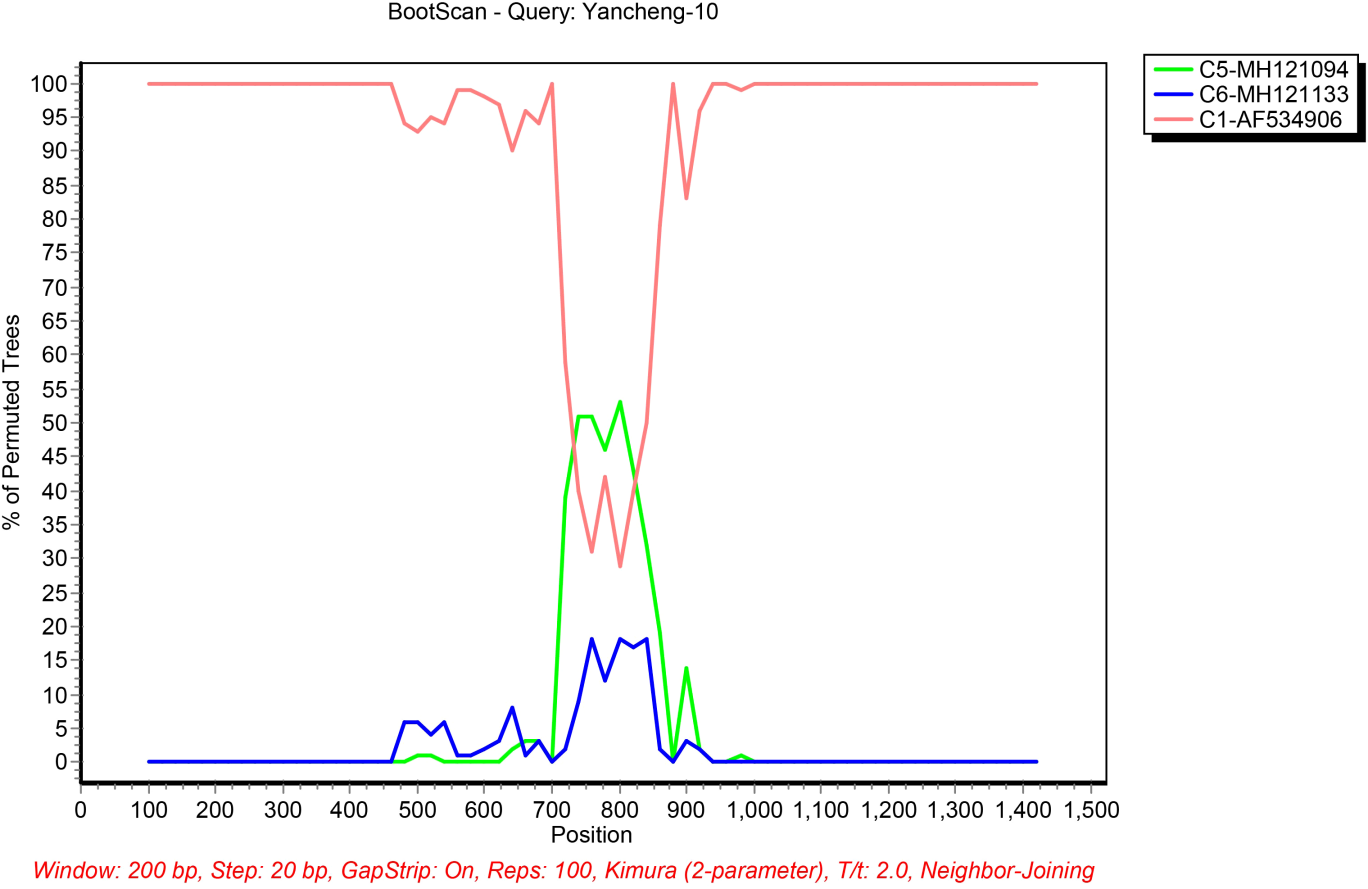
Bootscanning analysis of the HAdV sequences using a sliding window of 200 nt moving in 20-nt steps. For each bootscanning analysis, the names of viruses of the query sequence were indicated in the plot.

The putative recombinant sites were further validated using SimPlot software with BootScan analysis (**Figure 4**). C1-AF534906 is likely the backbone of Yancheng-10, and C5-MH121094 and C6-MH121113 are potential genetic constituents. Recombination breakpoints were precisely mapped to nucleotide positions 720 and 840 in the Fiber gene between C1-AF534906 and C5-MH121094. For Yancheng-11, Simplot analysis revealed high sequence similarity to B21-AY601633, with no additional recombination signals detected.

## 4 Discussion

In this study, 37 of the 170 samples were unable to successfully amplify three target genes simultaneously. Therefore, 133 HAdV-positive samples from children were genotyped in Yancheng in winter of 2023-2024. 129 samples were identified as type B3, two as type B21, one as type C1, and one as type C5, indicating that HAdV-B3 was the dominant strain, followed by HAdV-B21, HAdV-C1, and HAdV-C5. HAdV-B3 is the most prevalent type worldwide, with outbreaks reported in nearly every country (Lynch and Kajon, 2016).

From 2004 to 2006, 34.6% of patients with ARI in the United States were caused by HAdV-3 (Wo et al., 2015). In China, HAdV-3 is endemic in northern, northwestern, and southern regions. From 2007 to 2010, 20.1% of severe acute respiratory infection (SARI) cases in Beijing were HAdV-positive, of which 19.1% were attributed to HAdV-3 (Li et al., 2015). 78.4% of HAdV-positive SARI cases in children were caused by HAdV-3 in Lanzhou from 2006 to 2009 (Jin et al., 2013). HAdV accounted for 12.0% of SARI cases, with HAdV-3 responsible for 70.0% in Guangzhou between 2012 and 2013 (Chen et al., 2016). Outbreaks of HAdV-3-associated respiratory diseases have been reported in China, such as pharyngeal conjunctivitis outbreaks in Shanghai and Shenzhen in 2002 (Duan and Xie, 2018), and an outbreak of febrile respiratory illness and pharyngeal conjunctival fever in Hangzhou in 2011 (Xie et al., 2012). HAdV-1 and HAdV-5, belonging to species C, are also common respiratory disease-causing strains. Although globally prevalent, they exhibit lower virulence and milder clinical manifestations compared to HAdV-3 (Arnberg et al., 2000). In China, HAdV-1 and HAdV-5 infections are widespread but do not dominate circulating strains. Among 552 ARI patients, 76 (13.8%) were HAdV-positive, including 6 cases of HAdV-1 and 1 case of HAdV-7 in Zhejiang between 2007 and 2010 (Li et al., 2015). To date, no outbreaks caused by HAdV-1 or HAdV-5 have been reported in China.

In this study, the B3 strain was the most prevalent strain in Yancheng in the winter of 2023-2024. As described above, for many years, the B3 strain has been the main prevalent strain in China, with high sequence conservation. Our findings are consistent with a study conducted in 2021, which reported the genetic identity and homology of HAdV-3 strains prevalent in China between 2014 and 2018 (Duan et al., 2021). Our research has confirmed that HAdV-3 strains exhibit a high conservation throughout the evolution, which may underlie its stable prevalence.

The analysis of amino acids variant sites identified several amino acid substitutions across the three target genes. Some mutations were located in the hypervariable regions (HVRs) of the Hexon protein, which are critical areas for the interaction between the virus and the host immune system. Mutations such as D144N and G146E, D265E/K and G266E may alter the charge distribution of HVRs, affecting the virus’s ability to bind to antibodies. G141R/Q and R267K may affect the structural stability of Hexon protein, indirectly affecting its binding ability to receptors. Structural changes may make it easier or harder for viruses to bind to host cell receptors. Mutations such as A269F and T445A of Hexon may increase the persistence of the virus in the host’s body, thereby improving its transmission efficiency (Lu et al., 2014; Pfortmueller et al., 2019). Although the mutation site is not located at the Fiber knob region (amino acids 479-497) - which contains the receptor-binding domain (e.g., CAR/Coxsackie-Adenovirus Receptor) - it may still influence receptor-binding efficiency through allosteric effects (Bergelson et al., 1997). The mutation of the Penton is also not located at the RGD (Arg Gly Asp) region, which binds to the host cell surface α v integrins (such as α v β 3 and α v β 5) to mediate virus entry into the host cell, but alters the structure of the virus RGD (Wickham et al., 1993). The specific effects of these mutations on adenovirus still need to be verified through further experiments.

The fiber protein is an essential component of the adenoviral capsid whose structure determines viral binding affinity to host cell receptors. In this study, the recombination event observed in Yancheng-10 strain at positions 720–840 are likely to disrupt the normal folding and stability of the fiber protein, potentially modifying its tertiary structure (Fang et al., 2024). Such structural changes could affect the binding affinity between the fiber protein and host cell surface receptors, consequently influencing viral infectivity. A limitation of this study is that only the Hexon, Penton, and Fiber genes were analyzed. Without whole-genome sequencing, we cannot completely exclude the possibility of recombination or mutations in other regions of the genome.

The relatively low prevalence of HAdV-B21, HAdV-C1, and HAdV-C5 strains may be attributed to amino acid substitutions that potentially impair viral fitness, including possible replication deficiencies or reduced host adaptability. However, the functional consequences of these genetic variations require further investigation. Specifically, the potential impacts on viral transmission dynamics, pathogenicity, and receptor-binding affinity remain undetermined due to current study limitations.

Conclusion, the epidemiological characteristics and clinical characteristics of HAdV infection were analyzed in this study. Molecular evolution analysis based on divergent sequences of Penton base, Hexon, and Fiber genes revealed that HAdV-B3 was the dominant strain in Yancheng, China, during the winter of 2023–2024, followed by HAdV-B21, HAdV-C1, and HAdV-C5. This study focused on adenovirus in influenza-negative ILI cases without considering coinfection. In the future, we will analyze the impact of adenovirus coinfection with other viruses on the condition. The study’s limitations include insufficient clinical metadata and a restricted sample size (especially for B21/C1/C5), limiting the analysis of genotype-phenotype correlation of B21/C1/C5 strains. The short collection period (less than one year) may also bias prevalence estimates. Expanded longitudinal surveillance with clinical linkage is needed.

## Data Availability

The datasets presented in this study can be found in online repositories. The names of the repository/repositories and accession number(s) can be found in the article/**Supplementary Material**.

## References

[B1] Arnberg N. Edlund K. Kidd A. H. Wadell G. (2000). Adenovirus type 37 uses sialic acid as a cellular receptor. J. Virol. 74, 42–48. doi: 10.1128/JVI.74.1.42-48.2000 10590089 PMC111511

[B2] Avendaño Carvajal L. Perret Pérez C. (2020). Epidemiology of Respiratory Infections. Pediatric Respiratory Diseases 1, 263–72. doi: 10.1007/978-3-030-26961-6_28

[B3] Bergelson J. M. Cunningham J. A. Droguett G. Kurt-Jones E. A. Krithivas A. Hong J. S.. (1997). Isolation of a common receptor for Coxsackie B viruses and adenoviruses 2 and 5. Science. 275, 1320–1323. doi: 10.1126/science 9036860

[B4] Brini I. Guerrero A. Ezzine I. K. Höller D. O. Hetzer B. Würzner R.. (2020). Human adenoviruses associated with respiratory illness in neonates, infants, and children in the Sousse area of Tunisia. J. Med. Virol 6, 145–151. doi: 10.1002/jmv.26375

[B5] Chang A. B. Harrhy V. A. Simpson J. Masters I. B. Gibson P. G. (2002). Cough, airway inflammation, and mild asthma exacerbation. Arch. Dis. Child. 86, 270–275. doi: 10.1136/adc.86.4.270 11919102 PMC1719138

[B6] Chen Y. Liu F. Wang C. Zhao M. Deng L. Zhong J.. (2016). Molecular identification and epidemiological features of human adenoviruses associated with acute respiratory infections in hospitalized children in southern China, 2012-2013. PloS One 11, e0155412. doi: 10.1371/journal.pone.0155412 27171486 PMC4865050

[B7] Chretien J. Holland W. Macklem P. Murray J. Woolcock A. (1984). Acute respiratory infections in children A global public-health problem. N Engl. J. Med. 310, 982–984. doi: 10.1056/NEJM198404123101509 6700693

[B8] Crawford-Miksza L. Schnurr D. P. (1996). Analysis of 15 adenovirus hexon proteins reveals the location and structure of seven hypervariable regions containing serotype-specific residues. J. Virol. 70, 1836–1844. doi: 10.1128/JVI.70.3.1836-1844.1996 8627708 PMC190011

[B9] Dou Y Li Y Ma C Zhu H Du J Liu H. (2018). Rapid diagnosis of human adenovirus B, C and E in the respiratory tract using multiplex quantitative polymerase chain reaction. Mol Med Rep 18 (3), 2889–2897. doi: 10.3892/mmr.2018.9253 PMC6102718 30015894

[B10] Duan Y Xie Z (2018). Epidemiological Overview of the Genotypes of Human Adenoviruses Causing Respiratory Infections in China. Chinese Journal of Experimental and Clinical Virology 32 (4), 430–434. doi: 10.3760/cma.j.issn.1003-9279.2018.04.022

[B11] Duan Y. Xu B. Li C. Bao Y. An S. Zhou Y.. (2021). Molecular characteristics of human adenovirus type 3 circulating in parts of China during 2014-2018. Front. Microbiol. 12. doi: 10.3389/fmicb.2021.688661 PMC8276179 34267738

[B12] Fang B. Lai J. Liu Y. Liu L. L. Yu X. Li X.. (2024). Hybrid sequencing for detailed genetic characterization of human adenoviruses. Sci. Rep. 14 (1), 29490. doi: 10.1038/s41598-024-78960-9 39604421 PMC11603307

[B13] Garnett C. T. Talekar G. Mahr J. A. Huang W. Zhang Y. Ornelles D. A.. (2009). Latent species C adenoviruses in human tonsil tissues. J. Virol. 83, 2417–2428. doi: 10.1128/JVI.02392-08 19109384 PMC2648257

[B14] Huijskens E. G. Biesmans R. C. Buiting A. G. Obihara C. C. Rossen J. W. (2012). Diagnostic value of respiratory virus detection in symptomatic children using real-time PCR. Virol J. 9, 276. doi: 10.1186/1743-422X-9-276 23164039 PMC3511061

[B15] Ismail A. M. Lee J. S. Lee J. Y. Singh G. Dyer D. W. Seto D.. (2018). Adenoviromics: mining the human adenovirus species D genome. Front. Microbiol. . doi: 10.3389/fmicb.2018.02178 PMC6141750 30254627

[B16] Jin Y. Zhang R. F. Xie Z. P. Yan K. L. Gao H. C. Song J. R.. (2013). Prevalence of adenovirus in children with acute respiratory tract infection in Lanzhou. China. Virol J. 10, 271. doi: 10.1186/1743-422X-10-271 23984826 PMC4015357

[B17] Kenmoe S. Vernet M. A. Le Goff J. Penlap V. B. Vabret A. Njouom R. (2018). Molecular characterization of human adenovirus associated with acute respiratory infections in Cameroon from 2011 to 2014. Virol J. 15, 153. doi: 10.1186/s12985-018-1064-x 30285778 PMC6171299

[B18] Lange C. E. Niama F. R. Cameron K. Olson S. H. Aime Nina R. Ondzie A.. (2019). First evidence of a new simian adenovirus clustering with Human mastadenovirus F viruses. Virol J. 16, 147. doi: 10.1186/s12985-019-1248-z 31775793 PMC6880561

[B19] Lei Y. Zhuang Z. Liu Y. Tan Z. Gao X. Li X.. (2023). Whole genomic sequence analysis of human adenovirus species C shows frequent recombination in tianjin, China. Viruses. 15, 1004. doi: 10.3390/v15041004 37112985 PMC10142000

[B20] Li Y. Zhou W. Zhao Y. Wang Y. Xie Z. Lou Y.. (2015). Molecular typing and epidemiology profiles of human adenovirus infection among paediatric patients with severe acute respiratory infection in China. PloS One 10, e0123234. doi: 10.1371/journal.pone.0123234 25856575 PMC4391708

[B21] Lion T. (2014). Adenovirus infections in immunocompetent and immunocompromised patients. Clin. Microbiol Rev. 27, 441–462. doi: 10.1128/CMR.00116-13 24982316 PMC4135893

[B22] Liu L. Qian Y. Han Z. Jia L. Dong H. Zhao L.. (2023). Genetic evolution and variation of human adenovirus serotype 31 epidemic strains in beijing, China, during 2010-2022. Viruses. 15, 1240. doi: 10.3390/v15061240 37376540 PMC10305296

[B23] Lu Q. B. Tong Y. G. Wo Y. Wang H. Y. Liu E. M. Gray G. C.. (2014). Epidemiology of human adenovirus and molecular characterization of human adenovirus 55 in China, 2009-2012. Influenza Other Respir. Viruses. 8, 302–308. doi: 10.1111/irv.12232 24467816 PMC4181478

[B24] Lynch J. P. 3rd Fishbein M. Echavarria M. (2011). Adenovirus. Semin. Respir. Crit. Care Med. 32, 494–511. doi: 10.1055/s-0031-1283287 21858752

[B25] Lynch J. P. 3rd Kajon A. E. (2016). Adenovirus: epidemiology, global spread of novel serotypes, and advances in treatment and prevention. Semin. Respir. Crit. Care Med. 37, 586–602. doi: 10.1055/s-0036-1584923 27486739 PMC7171713

[B26] Madisch I. Harste G. Pommer H. Heim A. (2005). Phylogenetic analysis of the main neutralization and hemagglutination determinants of all human adenovirus prototypes as a basis for molecular classification and taxonomy. J. Virol. 79, 15265–15276. doi: 10.1128/JVI.79.24.15265-15276.2005 16306598 PMC1316018

[B27] Martin D. P. Murrell B. Golden M. Khoosal A. Muhire B. (2015). RDP4: Detection and analysis of recombination patterns in virus genomes. Virus Evol. 1, vev003. doi: 10.1093/ve/vev003 27774277 PMC5014473

[B28] Pan D. Zheng J. Chen Q. Zeng L. E. Lin C. You Y.. (2023). Clinical characteristics and genotyping of pediatric adenovirus pneumonia disease and coinfection in southeast China. Genet. Test Mol. Biomarkers. 27, 306–316. doi: 10.1089/gtmb.2023.0037 37768330

[B29] Pfortmueller C. A. Barbani M. T. Schefold J. C. Hage E. Heim A. Zimmerli S. (2019). Severe acute respiratory distress syndrome (ARDS) induced by human adenovirus B21: Report on 2 cases and literature review. J. Crit. Care 51, 99–104. doi: 10.1016/j.jcrc.2019.02.019 30798099 PMC7172394

[B30] Rowe W. P. Huebner R. J. Gilmore L. K. Parrott R. H. Ward T. G (1953). Isolation of a cytopathogenic agent from human adenoids undergoing spontaneous degeneration in tissue culture. Proc. Soc. Exp. Biol. Med. 84, 570–573. doi: 10.3181/00379727-84-20714 13134217

[B31] Shieh W. J. (2022). Human adenovirus infections in pediatric population - An update on clinico-pathologic correlation. BioMed. J. 45, 38–49. doi: 10.1016/j.bj.2021.08.009 34506970 PMC9133246

[B32] SSan Martín C. (2012). Latest insights on adenovirus structure and assembly. Viruses 4, 847–877. doi: 10.3390/v4050847 22754652 PMC3386624

[B33] Tamura K. Dudley J. Nei M. Kumar S. (2007). MEGA4: Molecular Evolutionary Genetics Analysis (MEGA) software version 4.0. Mol. Biol. Evol. 24, 1596–1599. doi: 10.1093/molbev/msm092 17488738

[B34] Walsh M. P. Chintakuntlawar A. Robinson C. M. Madisch I. Harrach B. Hudson N. R.. (2009). Evidence of molecular evolution driven by recombination events influencing tropism in a novel human adenovirus that causes epidemic keratoconjunctivitis. PloS One 4, e5635. doi: 10.1371/journal.pone.0005635 19492050 PMC2685984

[B35] Wang J Feng Q Duan Y Ai J Zhu Y Wang R. (2024). Human adenovirus type 4 (HAdV-4) associated acute respiratory tract infection in children & genetic characteristics of HAdV-4 in China: a prospective multicenter study. BMC Infect Dis. 24 (1), 936. doi: 10.1186/s12879-024-09835-7 PMC11385803 39251906

[B36] Weber M. W. Mulholland E. K. Greenwood B. M. (1998). Respiratory syncytial virus infection in tropical and developing countries. Trop. Med. Int. Health 3, 268–280. doi: 10.1046/j.1365-3156.1998.00213.x 9623927

[B37] Wickham T. J. Mathias P. Cheresh D. A. Nemerow G. R. (1993). Integrins αvβ3 and αvβ5 promote adenovirus internalization but not virus attachment. Cell. 73, 309–319. doi: 10.1016/0092-8674(93)90231-E 8477447

[B38] Wo Y Lu QB Huang DD Li XK Guo CT Wang HY. (2015). Epidemical features of HAdV-3 and HAdV-7 in pediatric pneumonia in Chongqing, China. Arch Virol. 160 (3), 633–8. doi: 10.1007/s00705-014-2308-8 PMC7087000 25504360

[B39] Wu X. Zhang J. Lan W. Quan L. Ou J. Zhao W.. (2022). Molecular typing and rapid identification of human adenoviruses associated with respiratory diseases using universal PCR and sequencing primers for the three major capsid genes: penton base, hexon, and fiber. Front. Microbiol. 13. doi: 10.3389/fmicb.2022.911694 PMC9133664 35633710

[B40] Xie L. Yu X. F. Sun Z. Yang X. H. Huang R. J. Wang J.. (2012). Two adenovirus serotype 3 outbreaks associated with febrile respiratory disease and pharyngoconjunctival fever in children under 15 years of age in Hangzhou, China, during 2011. J. Clin. Microbiol. 50, 1879–1888. doi: 10.1128/JCM.06523-11 22442311 PMC3372113

[B41] Xie L. Zhang B. Zhou J. Huang H. Zeng S. Liu Q.. (2018). Human adenovirus load in respiratory tract secretions are predictors for disease severity in children with human adenovirus pneumonia. Virol J. 15, 123. doi: 10.1186/s12985-018-1037-0 30086789 PMC6081882

[B42] Yao L. H. Wang C. Wei T. L. Wang H. Ma F. L. Zheng L. S. (2019). Human adenovirus among hospitalized children with respiratory tract infections in Beijing, China, 2017-2018. Virol J. 16, 78. doi: 10.1186/s12985-019-1185-x 31196108 PMC6567909

[B43] Yu J. Zhao S. Rao H. (2021). Molecular characterization of human respiratory adenoviruses infection in Xining city, China in 2018. Virol Sin. 36, 545–549. doi: 10.1007/s12250-020-00282-7 32926331 PMC8257811

[B44] Zou L. Yi L. Yu J. Song Y. Liang L. Guo Q.. (2021). Adenovirus infection in children hospitalized with pneumonia in Guangzhou, China. Influenza Other Respir. Viruses. 15, 27–33. doi: 10.1111/irv.12782 32761743 PMC7767961

