# Supplementary material for: Epidemiological characteristics of adenovirus in children in Yancheng, China, 2023-2024
Source: Front Cell Infect Microbiol. 2025 May 29;15:1587257. doi: 10.3389/fcimb.2025.1587257 (PMC12158925; doi:10.3389/fcimb.2025.1587257)
Supplement: Supplementary file 1 [file Table1.docx]

Supplementary Material

# Supplementary Figures and Tables

**Supplementary Table S1.** The chi-squared test for each clinical symptom between HAdV-positive patients and HAdV-negative patients.

| Clinical symptoms | HAdV cases  (n=170) | non-HAdV cases  (n=657) | χ^2^ | *p*-value |
| --- | --- | --- | --- | --- |
| Fever | 170 (100%) | 652 (99%) | 0.343 | 0.589 |
| Cough | 92 (54%) | 235 (36%) | 19.021 | 0.000013 |
| Sore throat | 47 (28%) | 88 (13%) | 20.086 | 0.000007 |
| Body aches | 11 (6%) | 30 (4%) | 1.039 | 0.308 |

**Supplementary Figure S2.** Identity of nucleotide and amino acid 28 strains (A, C, and E are the nucleotides identity of Hexon, Penton base and Fiber genes. B, D, and F are the amino acids identity of Hexon, Penton base and Fiber genes)**.**


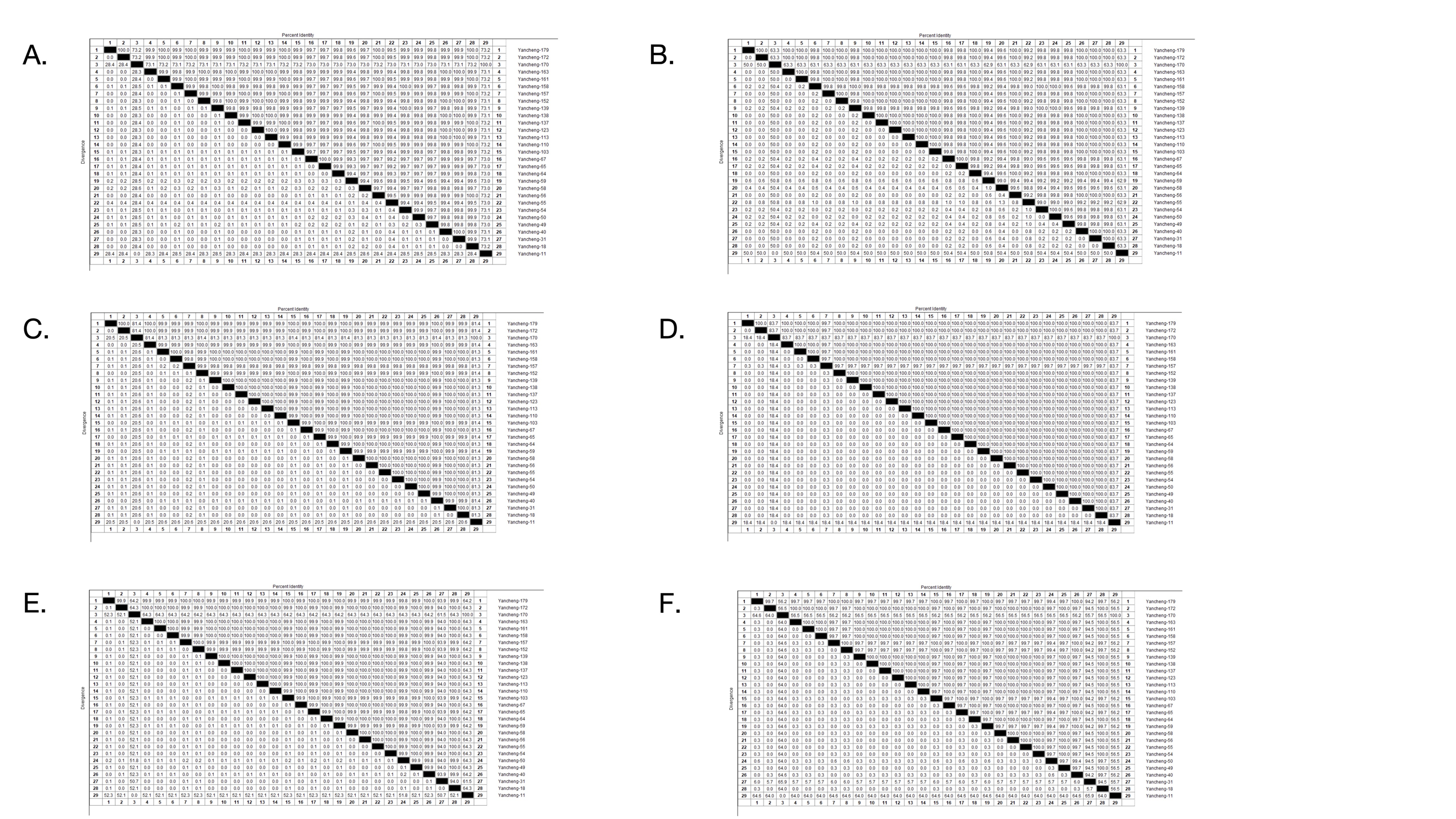


**Supplementary Table S3.** Nucleotide and amino acid Identities. (B21, B5, C1, and C5 denote the sample strains analyzed in this study.)

| Gene | Comparison | Nucleotide Identity (%) | Amino Acid Identity (%) |
| --- | --- | --- | --- |
| Hexon | B21 vs. B3 | 72.2-100 | 62.9-100 |
|  | B21 vs. AV-1645 | 99.1 | 98.1 |
|  | Among B3 strains | 99.4-100 | 99.0-100 |
|  | B3 vs. GB | 98.3-100 | 96.9-100 |
|  | C5 vs. C1 | 77.6 | 76.2 |
|  | C5 vs. AC_000008 | 99.2 | 99.6 |
|  | C1 vs. AF534906 | 99.9 | 99.9 |
| Penton | B21 vs. B3 | 81.3-100 | 83.7-100 |
|  | B21 vs. AV-1645 | 94.5 | 93.7 |
|  | Among B3 strains | 99.8-100 | 99.0-100 |
|  | B3 vs. GB | 98.0-100 | 98.9-100 |
|  | C5 vs. C1 | 96.1 | 96.8 |
|  | C5 vs. AC_000008 | 97.6 | 98.4 |
|  | C1 vs. AF534906 | 99.4 | 99.2 |
|  | All C types | 96.1-100 | 96.2-100 |
| Fiber | B21 vs. B3 | 61.5-100 | 55.7-100 |
|  | B21 vs. AV-1645 | 99.4 | 99.1 |
|  | Among B3 strains | 94.0-100 | 93.8-100 |
|  | B3 vs. GB | 92.6-100 | 93.1-100 |
|  | C5 vs. C1 | 75.6 | 50.4 |
|  | C5 vs. AC_000008 | 99.9 | 99.8 |
|  | C1 vs. AF534906 | 99.5 | 99.0 |
